# A Superpixel-Based Algorithm for Detecting Optical Density Changes in Choroidal Optical Coherence Tomography Images of Diabetic Patients

**DOI:** 10.3390/s25123619

**Published:** 2025-06-09

**Authors:** Sofia Otin, Victor Mallen-Gracia, Luis Perez-Maña, Francisco J. Ávila, Elena Garcia-Martin

**Affiliations:** 1 Department of Applied Optics, University of Zaragoza, 50009 Zaragoza, Spain; avila@unizar.es; 2 Miguel Servet University Hospital, 50009 Zaragoza, Spain; victormallen6@gmail.com (V.M.-G.); egmvivax@gmail.com (E.G.-M.); 3 Miguel Servet Ophthalmology Research Group (GIMSO), Aragon Health Research Institute (IIS Aragon), 50009 Zaragoza, Spain; 4 Department of Optics and Optometry, Polytechnic University of Catalunya, 08034 Barcelona, Spain; luis.perez.mana@upc.edu

**Keywords:** algorithm, OCT, diabetic, optical density

## Abstract

Background: This study explored the diagnostic potential of image-processing analysis in optical coherence tomography (OCT) images to detect systemic vascular changes in individuals with systemic diseases. Methods: Ocular OCT images from two cohorts diabetic patients and healthy control subjects were analyzed. A novel Superpixel Segmentation (SpS) algorithm was used to process these images and extract optical image density information from ocular vascular tissue. The algorithm was applied to isolate the choroid layer for analysis of its optical properties. The procedure was performed by separate examiners, and both inter- and intra-observer repeatability were assessed. Choroidal area (CA) and choroidal optical image density (COID) metrics were used to assess structural changes in the vascular tissue and predict alterations in the choroidal parameters. Results: A total of 110 diabetic patient eye images and 92 healthy control images were processed. The results showed significant differences in CA and COID between diabetic and healthy eyes, indicating that these parameters could serve as valuable biomarkers for early vascular damage. Conclusions: The use of the SpS algorithm on OCT B-scan images allows for the identification of new parameters linked to ocular vascular damage. These findings suggest that digital image-processing techniques can reveal differences in vascular tissue, offering potential new indicators of pathology.

## 1. Introduction

Digital image-processing encompasses techniques and algorithms designed to enhance, analyze, and extract semantic information from digital images. This field has become a fundamental tool in various scientific disciplines, particularly medicine. An emerging branch within digital image-processing is the application of machine learning (ML), a sub-field of artificial intelligence that enables systems to recognize complex patterns and make predictions based on image data. These techniques hold vast potential for the analysis of medical images, such as those obtained using optical coherence tomography (OCT).

OCT is a noninvasive imaging technique that provides high-resolution visualization of ocular structures, delivering detailed cross-sectional views of the retina and choroid. The integration of spectral domain OCT and swept-source OCT (SS-OCT) technologies has significantly enhanced the capability to evaluate deep tissues like the choroid, which play a critical role in various ocular and systemic pathologies.

The combination of advanced image-processing techniques and ML algorithms opens up new avenues for in-depth image analysis, enabling identification of potential markers that signal structural alterations, particularly in vascular physiopathology, as characterized by macroangiopathy and microangiopathy resulting from alterations caused by pericyte loss and capillary drop-out. For instance, diabetes mellitus (DM) can lead to microvascular changes that affect both the retinal and choroidal blood vessels, often well before the onset of diabetic retinopathy (DR) [1].

Recent studies have demonstrated changes in choroidal vascular density and structure in patients with DM who do not yet exhibit DR, underscoring the importance of this layer in evaluating early complications [2,3,4]. Similarly, other systemic diseases, such as neurodegenerative conditions, show vascular wall damage that can be detected using OCT [5].

The analysis of OCT B-scans through digital processing enables segmentation of the retinal layers, including the choroid, and facilitates the examination of information within regions that share similar image characteristics. This approach allows for the quantification of parameters, such as tissue thickness and the optical density of vascular structures. Considering these advances, our study endeavored to harness the capabilities of AI as applied to ophthalmic imaging. Specifically, we aimed to implement an algorithm in a cross-sectional study that sought to interpret image information derived from OCT B-scans obtained from subjects.

This study sought to detect subtle alterations in choroidal signal absorption (expressed as semantic differences in optical coherence tomography (OCT) scans) that elude standard ophthalmoscopic examinations. We define early detection as the evaluation of eyes from individuals with Type 2 diabetes who are still classified as ETDRS Level 10, i.e., completely free of clinically recognizable diabetic retinopathy. By analyzing this strictly preclinical stage (before the appearance of microaneurysms or other characteristic lesions), we sought to characterize the earliest vascular and stromal changes and determine whether superpixel-derived metrics, such as choroidal area (CA) and choroidal image optical density (COID), can serve as sensitive biomarkers of this transition. The proposed SpS algorithm is lightweight enough to run in real time on the OCT device’s embedded processor, integrating the analysis into the sensor itself and exemplifying smart sensor signal processing in line with the recent edge computing approaches described in *Sensors*. Finally, we demonstrate that the complete SpS workflow on OCT B-scan images offers high intra- and inter-observer repeatability, confirming its robustness and potential for routine clinical implementation.

## 2. Materials and Methods

### 2.1. Data Collection

To perform this study, it was necessary to obtain ocular OCT images, specifically B-scans centered on the fovea. The OCT images were obtained from volunteers who had participated in the “Retinal Alterations Diagnosis and OCT” study approved by the Aragon Research Ethics Committee in previous years; all participants were informed about the study’s details and provided informed consent. Specifically, subjects were obtained from among patients referred from the Department of Endocrinology who had been diagnosed with Type II DM, and healthy subjects were recruited from members of the Department of Ophthalmology and the companions of the patients. All volunteers were recruited between 2016 and 2020 and underwent comprehensive ophthalmological examinations, including a retinal examination using OCT technology, to confirm the absence of ocular pathology. At baseline (2016), patients with Type 2 diabetes had a mean age of 56.4 ± 8.2 years, 61% were male, their mean body mass index was 29.7 ± 3.4 kg/m^2^, and their diabetes duration averaged 5.05 ± 3.47 years. Seven years later (2023), 48% had LDL > 160 mg/dL, 88% hypertension, 94% remained overweight, and 40.7% fulfilled metabolic-syndrome criteria. Glycemic control worsened modestly (fasting glucose, 1.95 mg/dL; HbA1c, 0.21%, *p* < 0.001). Best-corrected visual acuity, intra-ocular pressure, and the dilated fundus examination remained within normal limits, and no eye developed clinical diabetic retinopathy (ETDRS 10). The healthy control group had a comparable age (55.9 ± 7.5 years), sex distribution (59% male), and BMI (26.1 ± 2.8 kg/m^2^).

A trained technician captured the retinal OCT images, first utilizing SS-OCT technology and the high-definition line examination protocol with the Tru-Track eye-tracking technology available on the Triton^®^ (Topcon Corporation, Tokyo, Japan) Deep Range Imaging (DRI) SS-OCT device. Secondly, the technician used Spectralis^®^ OCT (Heidelberg Engineering, Heidelberg, Germany) and the macular line protocol, which employs spectral domain technology and an enhanced deep-imaging system. The OCT examination was always performed at the same time of day due to the schedules defined in the study agenda, which made it possible to avoid choroidal changes not attributable to the study subject but to the physiological changes that occur during the day [6]. An ophthalmologist (Examiner 1), who was involved in the present study, extracted and recompiled OCT B-scan images considered as high quality under the OSCAR-IB criteria from the database of previous cohorts of ocular OCT B-scan images taken from DM patients and healthy subjects. To ensure that the examiners in charge of processing the images were blind to the features of the eyes (pathological or healthy), the photos were randomly numbered and exported in TIFF format at a resolution of 1024 × 992 pixels (px). Images from Spectralis^®^ OCT were used to assess the capability of SpS to detect and delineate choroidal boundaries. For subsequent image processing to extract optical density information, Triton^®^ OCT B-scan images were used to reduce bias.

### 2.2. Image-Processing Protocol

The Triton^®^ OCT B-scan images extracted were processed using the main kernel of Superpixel Segmentation imaging. The superpixel method joins pixels with comparable structural qualities to produce clusters with similar meaningful properties, called superpixels. The number of superpixels, the number of iterations, and the regularity of the shape of the detected superpixels are predefined by the specialist and set prior to the following steps.

We developed a custom interactive Matlab^®^ (V. R2025a) script (Math Works^®^) for semiautomatic selection and quantitative analysis of choroidal regions from OCT imaging, based on superpixel segmentation. The processing pipeline can be summarized as follows.

*Step 1. Image Processing*. Let $I:\;\Omega \subset Z^{2}\to R$ be a grayscale OCT image defined over the spatial domain Ω. Then a sharpening filter is applied for edge enhancement using the defined Matlab function (‘imsharpen’):
  (1) $$I_{s}=imsharpen(I)$$
*Step 2. Superpixel Segmentation*. The image *Is* is partitioned into N non-overlapping superpixels using the Simple Linear Iterative Clustering (SLIC) algorithm
  (2) $$\left\{S_{1},S_{2},\ldots ,\;S_{n}\right\},\;{⋃}_{i=1}^{N}S_{i}=\Omega ,\;S_{i}⋂S_{j}=\emptyset ,\;\mathrm{for}\;i\neq j.$$
  where each superpixel *S_i_* corresponds to a set of connected pixels sharing a similar intensity and proximity.
*Step 3. Manual Superpixel Selection*. The user interactively selects a subset S⊂{1,…,N} of superpixels. A binary mask M: Ω→{0, 1} is constructed as:
  (3) $$M\left(x,y\right)=\left\{\begin{array}{c} 1\;if\;\left(x,y\right)\in \cup_{i\in S}\;S_{i}\\ 0\;otherwise \end{array}\right.$$
*Step 4. Morphological Smoothing*. To reduce boundary irregularities, morphological opening followed by closing is applied using a disk-shaped structuring element *B_r_* of the radius *r*
  (4) $$M_{s}=\left(M\circ B_{r}\right)\;●\;B_{r}$$
  where ∘ denotes morphological opening and ● denotes closing.
*Step 5a. Calculating the Total Area of the Smoothed Region*. The area of the smoothed region is calculated by summing all nonzero pixels in the mask.
*Step 5b. Visualizations*. Three visualizations are created: (A) the smoothed region alone; (B) the smoothed mask overlaid on the original image with semi-transparency; (C) the fluorescent green overlay of the smoothed area.

### 2.3. Precision and Accuracy

Three separate examiners (E) from different universities each independently processed the entire set of OCT B-scan images. In addition, one of the examiners (E2) repeated the processing on different days. Two of the examiners, E1 and E2, were specialists in vision and the ocular system; E3, however, was unaware of the purpose of the work. This latter examiner was only asked to select the areas they considered to be identical. Moreover, E3 had no prior knowledge of vision, the ocular system, or general pathology. Although SpS automatically processes the OCT B-scans, once the areas containing similar information are identified, they must be selected manually. A person knowledgeable in this area could unintentionally bias the selection of identical points if they were aware that it was the choroidal area that needed to be analyzed (superpixel selection Step 4).

### 2.4. Outcomes

To measure the detected structural alterations to the choroid, two metrics were established, namely choroidal area (CA), which is the total area corresponding to the segmented choroidal region of interest; within this area, choroidal optical image density (COID) was calculated, which is the mean pixel value of the segmented area.

### 2.5. Statistical Analysis

Statistical analyses were performed using the SPSS V.26 software (IBM SPSS Statistics. Chicago, Illinois, USA). The normality of the variables in the study was assessed using the Kolmogorov–Smirnov test (*p* > 0.05). Since the variables were considered to meet the criteria for normality, the subsequent comparative analysis used parametric tests. To explore the correlation between the CA measurements obtained using image processing with SpS and the Spectralis^®^ OCT device, the Pearson correlation coefficient was used. To assess the accuracy of the CA and COID value calculations under the semiautomatic method, inter-observer and intra-observer variability were analyzed using the coefficient of variation (CoV) and the intra-class correlation coefficient (ICC). To evaluate the potential to detect differences between healthy and pathological eyes, CA and COID values obtained via image processing were subjected to Student’s *t*-test.

## 3. Results

**Figure 1:**
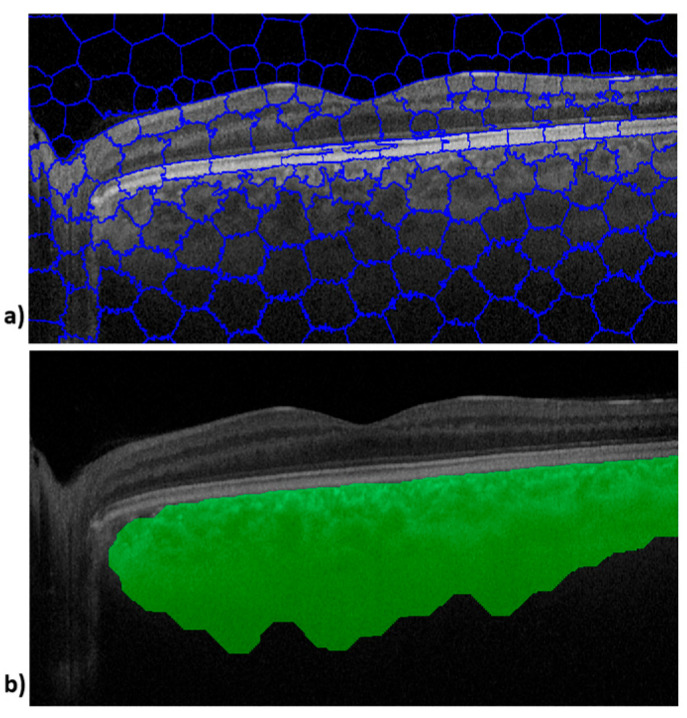
Image outputs obtained via Superpixel algorithm processing for one OCT B-scan image. The upper image (**a**) shows segmentation, and the lower image (**b**) shows the choroid tissue boundaries.

A total of 110 ocular OCT B-scans obtained from DM patients and 92 images obtained from healthy subjects were recompiled and randomly numbered by E1 before being sent for digital processing. All images were processed using SpS, and the CA and COID parameters were obtained from the segment selection performed by the three examiners. Figure 1 shows the image outputs obtained during processing of an OCT B-scan. The upper image (Figure 1a) shows SpS and the lower image shows the choroid tissue boundaries defined by the SpS algorithm (Figure 1b).

**Figure 2:**
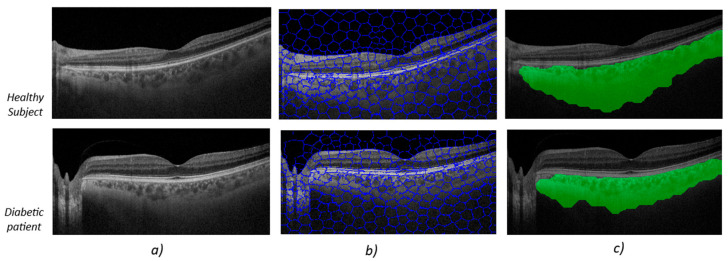
Image outputs obtained via Superpixel algorithm processing for one OCT B-scan image. The upper pictures are from healthy subjects, and the lower pictures are from diabetic patients. The left column (**a**) shows OCT B-scan images for both types of subject, the center column (**b**) shows segments with the same semantic information and the right column (**c**) shows choroid delimited tissue.

Figure 2 shows the image-processing output for two subjects chosen at random in each group. The upper image (Figure 2) shows the algorithm’s output for a healthy subject, while the lower image shows the algorithm’s output for a diabetes patient (Figure 2).

At first glance, the OCT B-scans (first column, Figure 2a) do not show any differences in the area corresponding to the choroidal tissue. It is important to remember that the image properties do not align with what the human eye might perceive as better image quality, such as greater contrast or sharpness. In the second column, the output after applying the SpS, which groups together areas of the image with the same semantic information, is noticeable. The third column (Figure 2c) contains the areas defined by SpS that correspond to zones with choroidal tissue.

The analysis using the algorithm-processing method revealed that the parameters were significantly different in eyes from healthy subjects than in those from diabetes patients (*p* < 0.001) as shown in Table 1. Optical image information, measured as COID, was much lower in diabetic subjects than in healthy eyes (*p* < 0.001). These results are consistent with those obtained in a study previously conducted in subjects with neurodegenerative disease [7].

## 4. Discussion

Various studies have consistently identified a reduction in choroidal thickness among individuals with DM, regardless of whether they manifest diabetic retinopathy or not [2,4,8]. In this regard, the availability of noninvasive tools with the potential to predict disease progression or facilitate early diagnosis is a crucial development. Such tools could not only aid early detection but could also play a pivotal role in population-wide screening and ongoing monitoring of the disease. Our research group is therefore endeavoring to apply the power of the Superpixel algorithm to choroidal analysis as a noninvasive biomarker for the early detection of systemic vascular changes in individuals with DM. Our efforts are founded on compelling scientific evidence that meticulous examination of the choroid allows an in-depth understanding of the microvasculature.

This study employed an innovative automated image-segmentation method to delineate the choroidal area and scrutinize the choroidal properties represented in OCT images as COID. Notably, this novel parameter has shown potential in vascular tissue evaluations of neurodegenerative patients’ conditions [7].

The density observed in optical images is related to the intensity of the light absorbed by the media on which the light is incident. On the basis of these values, the type of material that passes through light at a given point can be categorized. In a similar vein, our study of OCT images is concerned with the optical density of various structures. This understanding of optical density plays a pivotal role in image analysis and interpretation, allowing us to discern different features and materials. In the field of ophthalmology, the concept of optical image density has found applications in various domains, including anterior segment research. One notable instance is in corneal analysis using topography or OCT, where it has been shown that corneal optical image density exhibits an upward trend with natural aging, corneal degeneration, refractive surgery, and corneal transplants [9,10]. Importantly, this increase in optical image density is intrinsically linked to the preservation of corneal transparency, shedding light on the underlying mechanisms at play. Furthermore, the variations in corneal optical density have been associated with spectrum conditions, including keratinocyte inflammatory reactions and stromal fibrosis, emphasizing the role of optical image density as a valuable parameter in understanding the health and dynamics of the cornea [9,10]. Emerging studies are initiating the exploration of choroidal tissue analysis using OCT images, ushering in a highly promising avenue of research for the early detection of retinal abnormalities [11,12,13]. Nakano et al. found differences in the image density of diabetic subjects, with and without retinopathy, compared with healthy subjects, using a method similar to ours [14]. In DM patients, vessel walls are altered by chronic hyperglycemia, which leads to the loss of pericytes and ischemia of the vascular walls. Ischemia can also trigger fibrosis of the vascular walls in addition to vascular obstruction, neovascularization, or aneurysms [15,16]. The vascular change process associated with chronic hyperglycemia could be the reason for the increased optical density observed in OCT images. Significant differences were identified in all the choroidal parameters extracted from B-scans of the DM patients and healthy controls participating in this study: COID and choroidal area. In this context, a recent study applied superpixel-based analysis to laser speckle flowgraphy (LSFG) blood flow maps to assess how systemic factors like body weight, height, and sex affect ocular blood flow resistivity. The findings support the use of image-based metrics, such as BOM or COID, as noninvasive biomarkers of vascular health in ocular tissues [17].

This method could provide a useful new means of studying choroidal layer alterations applicable to the field of ophthalmology and beyond, to which end, we highlight some of the advantages and the potential impact of the evaluated parameters. The ability to identify alterations before retinal signs manifest could be crucial for early intervention and prevention of severe ocular complications. Although this study solely included eyes without diabetic retinopathy, it opens the possibility that the analyzed parameters may become future biomarkers for diabetic retinopathy. Long-term research may help determine if these parameters can predict the development of diabetic retinopathy. Choroidal layer parameters, such as COID, could be used as indicators of systemic vascular damage in DM patients.

The limitations of this study are as follows. (1) The sample size is relatively small, given the breadth of diabetic pathophysiology. Only subjects without progression to diabetic retinopathy were included; including a group in which this progression has occurred would allow us to determine whether the tool is useful for predicting the appearance of this complication. (2) It would be useful to analyze subgroups according to treatment received, glycosylated hemoglobin levels, and age ranges. This could be achieved by increasing the sample size to make these groups acceptable for subgroup analysis and would thus allow us to understand the behavior of choroidal vascular tissue in these scenarios. (3) It would be useful to validate the method in a population different from the one assessed in the study to examine its cost-effectiveness when applied to eyes of different genetic, racial, and environmental origin. External validation against manual delineations was not feasible because raw B-scan files were no longer accessible; nevertheless, CA values obtained with SpS correlate strongly with the manual ground truth reported in our previous study and with device-embedded segmentation. (4) An important limitation of the present work is its cross-sectional design, which prevents us from establishing whether the choroidal alterations observed truly precede, and therefore predict, the future development of diabetic retinopathy. A prospective longitudinal follow-up of this or a similar cohort tracking CA and COID trajectories alongside conventional retinal grading would be necessary to confirm the temporal sequence and to derive clinically meaningful prognostic cut-offs for the proposed SpS-OCT metrics. Such longitudinal validation, although desirable, lies beyond the scope of the current study.

## 5. Conclusions

The present cross-sectional findings suggest that CA and COID may prove useful as early indicators of choroidal change in diabetes; however, further external validation and longitudinal follow-up are required before clinical implementation. A key advantage of this method is its noninvasive technique, making it ideal for use in clinical settings. Additionally, OCT imaging is relatively cost-effective when compared with other diagnostic procedures. In summary, application of this choroidal analysis method shows potential in the detection of vascular ocular damage in DM patients and could extend to assessment of vascular damage in other ocular pathologies.

It is also a simple technique that can be implemented in the software of commercially available OCT devices and can be used in daily medical practice or even in population screening programs, in primary care, or endocrinology consultations due to its simplicity and ease of acquisition.

## Figures and Tables

**Table 1:** Comparison of variables between healthy and DM subjects using Student’s *t*-test. Significant differences are marked in bold.

Parameter		Diabetic Eyes		Healthy Eyes		*p*-Value
		Mean	SD	Mean	SD	
Image Processing (from Triton^®^ OCT B-scans)	CA (px^2^)	88,016.71	20,978.93	154,435.85	19,714.12	**<0.001**
	COID	71.88	9.09	57.47	6.024	**<0.001**
Spectralis^®^ OCT	CA (mm^2^)	1.62	0.61	2.11	0.15	**<0.001**

Abbreviations: SD, standard deviation; CA, choroidal area; px, pixel; COID, choroidal optical image density; mm, millimeter.

## Data Availability

The data presented in this study are not publicly available due to confidentiality restrictions; however, they are available from the corresponding author upon reasonable request.

## Abbreviations

The following abbreviations are used in this manuscript.

CA	Choroidal area
COID	Choroid optical image density
DM	Diabetes mellitus
OCT	Optical coherence tomography
SpS	Superpixel segmentation
